# The antiproliferative and apoptotic profile of gomesin against DFTD

**DOI:** 10.1038/s41419-018-0885-2

**Published:** 2018-08-06

**Authors:** Maria P. Ikonomopoulou, Manuel A. Fernandez-Rojo

**Affiliations:** 1 Madrid Institute for Advanced Studies (IMDEA) in Food, CEI UAM+CSIC, Madrid, E28049 Spain; 2 School of Medicine, The University of Queensland, Herston, QLD 4006 Australia; 3 Tranlastional Research Institute/Diamantina Institute, The University of Queensland, Herston, QLD 4006 Australia

Tasmanian devil is the largest remaining marsupial carnivore endemic in Tasmania. For the past 20 years it is driven to extinction owing to the devil facial tumour disease (DFTD), a transmittable (contagious) type of cancer^1^. This unique form of cancer is transmitted as an allograft during physical close contact (biding) among devils^1^. DFTD was first recorded in 1996 in the northeastern of Tasmania but since then it has been spread in almost all over the island. DFTD has been classified as two clone types: DFTD Type 1 and DFTD type 2, which have appeared at different geographical locations and chronological orders in Tasmania^2^. In both strains, large tumours appear first on the face and mouth and fast metastasise to internal organs, leading to the death of the animal within 6 months of infection.

**Fig. 1:**
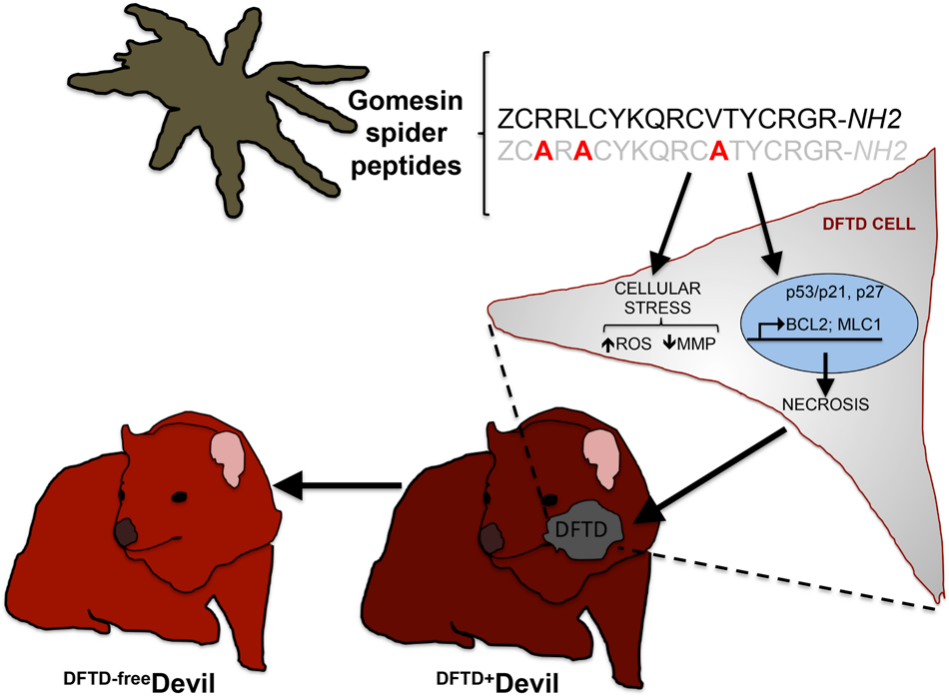
**Diagram showing the antiproliferative and apoptotic profile of gomesin-spider peptides in DFTD cells.** Gomesin peptides cause cellular stress owing to an increase in the cellular reactive oxygen species (ROS), a reduction of the mitochondrial membrane potential (MMP) and stimulation of the expression of p53, p21, p27, BCL2 and MLC1. The model postulates that all together lead to cellular necrosis and subsequent reduced DFTD cell viability. Gomesin cytotoxicity is prevented by alanine substitution in residues R3, L5 or V12. The model also suggests that gomesin may assist in the development of a drug candidate against DFTD and thus to a DFTD-free Tasmanian devil population

The Tasmanian devil genome exhibits high homology with the human genome, including ~ 88% similarity with various human cancer linked genes^3^. Approximately 17,000 somatic mutations have been reported in the DFTD genome, in comparison with the 5000 average mutation number exhibited by most of the human cancers, suggesting that DFTD has suffered several evolution cycles^3^. In addition, DFTD cells are characterised by a diverse number of tetraploid variants, a trend observed in other highly aggressive, metastatic and possibly drug-resistance cancer types^4^. The genetic signature of DFTD is defined by a pool of genes that includes myelin related genes (i.e., MPZ, PRX, MBP, PMP22) as well as transcription factors linked with the differentiation of schwann cells (i.e., SOX10, SOX2, POUSFI, JUN), NES, NGFR and S100^3^. Of interest are the oncogenes APC, MYC, NF2 and MLH1, which are suggested to be mutated and; thus, to play a role in DFTD tumourogenesis^5^. However, the lack of genetic diversity among the devil populations as well as the fact that DFTD cells do not express cell surface MHC molecules owing to the downregulation of antigen-processing pathway-linked genes, such as β2-microglobulin and transporters associated with antigen processing^6^ has been postulated to have contributed to the rapid expansion of the disease.

Until recently, the quest for a suitable chemotherapeutic agent against DFTD has been a neglected and obscure area of study. Indeed, the main approach for the conservation of the species has been on the development of prevention and management strategies for controlling the disease. Although there have been efforts to treat DFTD-infected devils with FDA-approved anti-cancer drugs such as vincristine, carboplatin and doxorubicin, the results have been unsuccessful^7,8^. To increase the therapeutic chances of success of novel compounds, researchers implement cell and molecular biology techniques to validate their anti-tumoral properties and prior to clinical or animal trials. Moving towards this direction, in a recently published study in Cell Death Discovery, Fernandez-Rojo et al.^9^ challenged the current dogma that DFTD cure lies only within prevention, genetic or immunological approaches and demonstrated that animal-derived compounds could emerge as a promising source of novel therapeutic compounds against infected devils (Fig. 1). This study thoroughly characterises the molecular mechanisms of the spider-peptide gomesin and its analogues to prevent the proliferation of DFTD cells^9^ by combining biochemistry, cell biology, gene expression, medicinal chemistry and computational modelling analyses. Furthermore, this study shed light on the associated molecular-fingerprint as well as on the specific amino acids responsible for the antiproliferative and apoptotic properties of gomesin in DFTD cells. These insights could assist in the design and development of novel and exponentially more potent and specific gomesin-derived compounds against DFTD. At a mechanistic level, Fernandez-Rojo et al.^9^ provide evidence that gomesin targets DFTD cells in a cell-type-specific manner, causing cell cycle arrest at G0/G1 phase. Cell proliferation is balanced between pro-proliferative and senescence signalling cascades. In tumour cells, this balance is disrupted; for example, owing to aberrant regulation of gene pathways that promote cell replication and mitosis, including the pro-survival and anti-apoptotic cascades. In DFTD cells, the antiproliferative signature of gomesin comprises stimulation of the p53/p21 cell cycle checkpoint axis as well as p27 expression, which have been shown to repress the progression through the G1 phase. Gomesin-induced cell cycle arrest is accompanied by bona fide hallmarks of cellular stress such as the generation of reactive oxygen species and a diminished mitochondrial membrane potential. Although initially gomesin might stimulate compensatory pro-survival responses as reflected by the elevated expression of BCL2 and MCL1, cellular stress eventually succumbs to unprogrammed cell death in the treated DFTD cells^9^. Furthermore, elaborated computational modelling suggests that the cytotoxicity occurs independently of changes in peptide structure, electrostatic potential surface and differences in conformation flexibility of gomesin. Of interest, single amino-acid substitutions exposed the key residues responsible of the antiproliferative activities of gomesins. Arginine substitutions in positions 8 and 9 (K8R and Q9R) enhanced the most the antiproliferative capacity, whereas alanine replacement in key residues 3, 5 or 12 (R3A, L5A or V12A) eradicated the cytotoxicity of gomesin. Another study by Pachette et al.^10^ showed that the immunomodulatory molecule imiquimod displays an apoptotic activity after prolonged exposure in DFTD cells. Therefore, gomesin and imiquimod may constitute the foundation for the design of chemotherapies that could be implemented into conservation and management strategies for the diseased Tasmanian devils. In addition, owing to the diversity, complexity and pharmacological potential of animal venoms and animal compounds^11^, it is possible that gomesin constitutes the first lead-candidate for the future development of novel chemotherapeutic drugs against DFTD.

## Conclusions

In summary, the study by Fernandez-Rojo et al. (2018) demonstrates that the spider peptide, gomesin, selectively inhibits the proliferation of devil facial tumour cells in vitro by manipulating the balance between pro-survival and pro-apoptotic pathway activities and key signalling cascades that control cell death and proliferation. Most importantly, they reveal the key amino acids that mediate cytotoxicity in gomesin. Consequently, this study provides the basis for further studies to evaluate and validate in vivo the therapeutic capabilities of gomesin against DFTD.
